# Erratum

**DOI:** 10.1111/jdi.13019

**Published:** 2019-03-05

The authors would like to draw the reader's attention to the errors in the following article.

Kusunoki‐Tsuji C, Araki SI, Kume S, Chin‐Kanasaki M, Osawa N, Morino K, Sekine O, Ugi S, Kashiwagi A, Maegawa H. Impact of obesity on annual medical expenditures and diabetes care in Japanese patients with type 2 diabetes mellitus. *J Diabetes Investig* 2018; 9: 776–781.

In Table 2 on page 779, hospitalization cost under ‘All’ column should have been ¥385,410 (¥253,910–779,120) and outpatient services cost except for medications under ≥25 column should have been ¥100,650 (¥57,120–188,895), as revised in the table below.

**Table 1:** Annual medical expenditures of all patients and of two subgroups stratified by obesity status

Variable	All	Body mass index (kg/m^2^)		*P*‐value†
		<25	≥25	
Total	¥269,333 (¥169,664–437,437)	¥252,263 (¥153,218–389,635)	¥294,548 (¥183,090–488,909)	0.004
All outpatient services‡	¥260,111 (¥163,671–400,038)	¥242,082 (¥151,866–356,663)	¥293,792 (¥181,923–448,031)	0.001
Outpatient services except for medications	¥95,900 (¥54,630–187,061)	¥92,490 (¥53,775–182,850)	¥100,650 (¥57,120–188,895)	0.22
Medications	¥133,183 (¥82,120–212,902)	¥120,663 (¥72,994–176,934)	¥161,931 (¥101,719–247,644)	<0.001
Hyperglycemia	¥67,127 (¥26,704–107,071)	¥60,439 (¥19,107–99,800)	¥79,647 (¥39,824–132,399)	<0.001
Hypertension	¥26,748 (¥0–60,996)	¥19,893 (¥0–53,338)	¥44,895 (¥1,149–72,578)	0.001
Dyslipidemia	¥18,382 (¥0–39,303)	¥9,888 (¥0–36,849)	¥24,354 (¥0–41,930)	0.02
Hospitalization§	¥385,410 (¥253,910–779,120)	¥307,119 (¥247,845–599,400)	¥602,079 (¥284,913–1,030,000)	0.03

Data are expressed as medians of Japanese yen (interquartile range). Obesity categorized by body mass index (BMI) ≥25 kg/m^2^. ^†^Differences between subgroups were compared using Mann–Whitney *U*‐tests for skewed continuous variables. ^‡^All outpatient services = total medical expenditures – hospitalization costs. ^§^Data were analyzed only in patients that were hospitalized (total 54 patients; 31 without and 23 with obesity).

The authors apologize for these errors and any confusion they may have caused.

